# Using a NanoBRET‐Based Ligand‐Binding Assay at the β_2_‐Adrenoceptor for Undergraduate Pharmacology Education

**DOI:** 10.1002/prp2.70275

**Published:** 2026-06-09

**Authors:** Abigail Pearce, Xianglin Huang, Theo Redfern‐Nichols, Claudia M. Sisk, Dewi Safitri, Michael Collins, Milena A. Malcharek, Sergio Tomey García, Chiharu Nakamura, Andrzej Harris, Graham Ladds

**Affiliations:** ^1^ Department of Pharmacology University of Cambridge Cambridge UK; ^2^ Department of Molecular and Clinical Cancer Medicine, Institute of Systems, Molecular and Integrative Biology University of Liverpool Liverpool UK; ^3^ School of Pharmacy Insititut Teknologi Bandung Bandung Indonesia

**Keywords:** β_2_‐adrenoceptor, G protein‐coupled receptor, *K*_d_, NanoBRET binding, teaching, undergraduate

## Abstract

The teaching of pharmacology includes a fundamental understanding of ligand binding; how it is measured and how it is calculated. We sought to modernize the techniques by which ligand affinity is determined by undergraduate students and introduce them to parameter estimation through curve fitting. We taught a NanoBRET ligand binding assay to student cohorts completing their second year of undergraduate study in Natural Sciences (71 and 61 students in 2024 and 2025 respectively). The aim was to measure the affinity of fluorescent and unlabeled ligands for the β_2_‐adrenoceptor in live cells. Affinities were then calculated from their data using a custom Microsoft Excel spreadsheet. This series of practical classes was well received by students, with most students able to follow the protocol and successfully determine ligand affinities. Furthermore, students recognized the benefit of the practical class for their education, confirming they felt it improved their understanding of how ligand affinity is calculated. We also demonstrated that this protocol could be scaled up to accommodate larger class sizes (class of 367 students studying medicine and veterinary sciences).

**Abbreviations**
β_2_AR: β_2_‐adrenoceptor
AA: antibiotic‐antimycotic
BSA: bovine serum albumin
DMEM: Dulbecco's modified Eagle's medium
DMSO: dimethyl sulfoxide
FBS: fetal bovine serum
GPCR: G protein‐coupled receptor
*K*_d_
: dissociation constant
*K*_i_
: inhibition constant
MoDA: mechanism of drug action
NanoBRET: NanoLuc bioluminescence resonance energy transfer
NLuc: NanoLuc
NST: natural sciences tripos
PBS: phosphate‐buffered saline

## 1 Introduction

Pharmacology is the study of how drugs and medicines interact with the body, comprising pharmacokinetics and pharmacodynamics. The latter focuses on determining how medicines act across organs and tissues to exert therapeutic and potential toxic effects. Pharmacodynamics can offer quantitative information on the concentrations across which a drug binds its target (affinity—e.g., *K*_d_) and exerts its effects (e.g., pEC_50_ and pIC_50_). Understanding how these metrics are calculated is an important aspect of any taught course in pharmacology [1, 2], forming part of the core knowledge statement “Theoretical principles of drug action” as described by the British Pharmacological Society (BPS) (https://www.bps.ac.uk/careers‐education/teaching‐pharmacology/undergraduate‐curriculum/). Practical classes can be employed to enhance this theoretical learning, reinforcing ideas as well as providing core skills (e.g., BPS undergraduate curriculum—“Experimental Techniques”).

Measuring agonist and antagonist affinity and efficacy is often performed using ex vivo animal tissue. Whilst informative, ex vivo experiments are associated with many disadvantages. Due to the variation of the tissue, results are often non‐reproducible and unreliable. Furthermore, as in many areas of scientific research, and pertinently within the University of Cambridge and scientific community within the United Kingdom, efforts are being made to reduce and replace animal usage [3]. As such, these experiments no longer mirror the tools used within academic and industrial settings, which instead favor more technologically advanced techniques that can be adapted for higher throughput screening. By updating this protocol, we further align with the BPS core curriculum of methodological principles, with the study of emerging methods to interrogate pharmacodynamics, and the tools used in scientific research and drug development.

**FIGURE 1:**
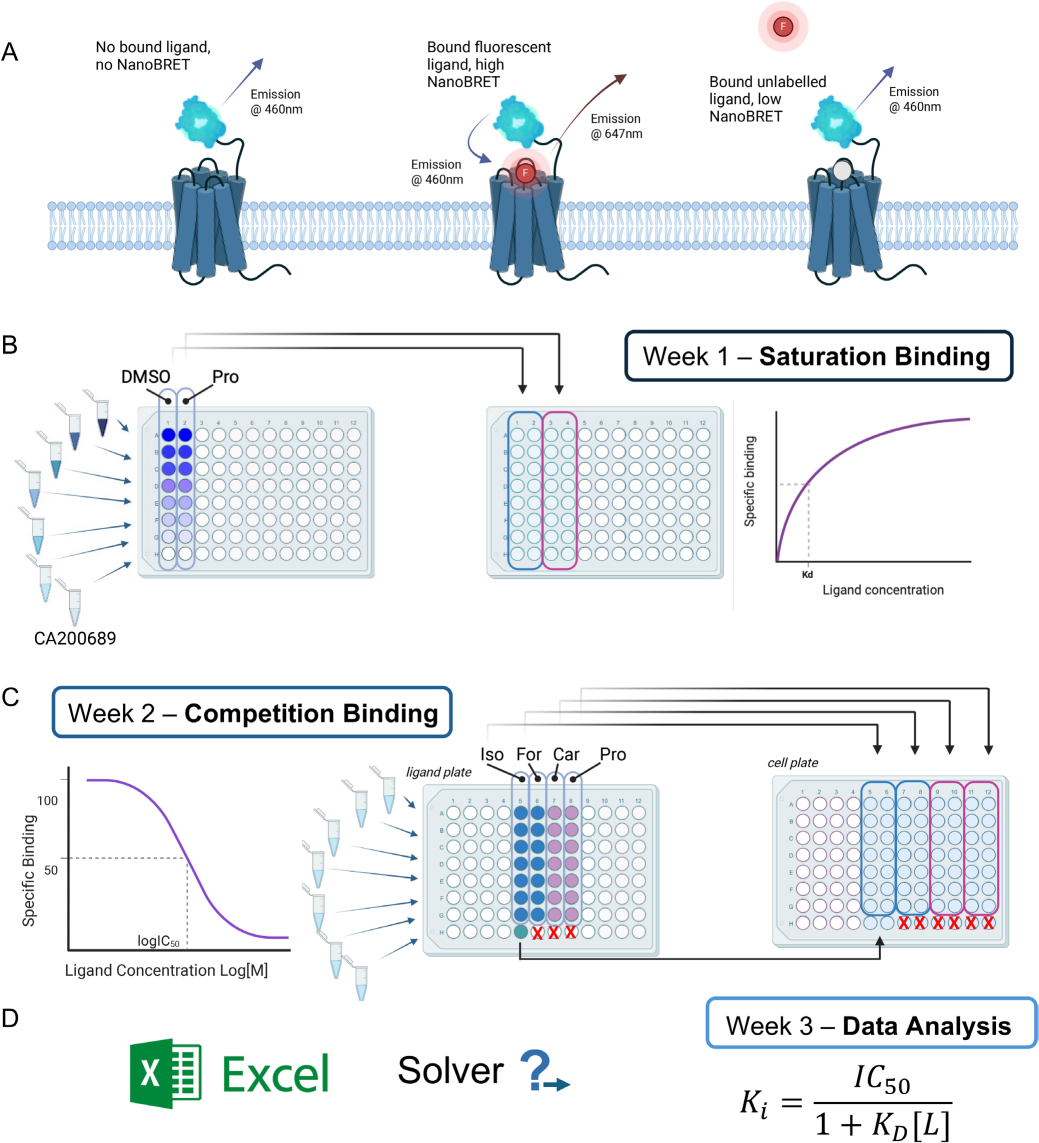
A three‐part practical to determine ligand affinity with NanoBRET binding. (A) Schematic of NanoBRET ligand binding, for measuring affinities at GPCRs. (B) Summary of the protocol for Saturation Binding, completed in the first session. (C) Summary of the protocol for Competition Binding, completed in the second session. (D) Data Analysis session using the Solver function in Microsoft Excel, and implementation of the Cheng‐Prusoff equation. Created in BioRender. Ladds, G. (2026) https://BioRender.com/tf4o9mg.

The G protein‐coupled receptor (GPCR) superfamily is one of the most therapeutically relevant classes of proteins, being the target of 36% of all approved drugs [4]. With frequent identification of new therapeutic indications, this class of proteins is unlikely to fade into obsolescence anytime soon and is thus relevant to incoming scientists. At the University of Cambridge, affinity calculations were previously obtained in undergraduate practical classes using radioligand binding, with preparations coming from homogenized guinea pig brains. Whilst radioligand binding studies can be completed in cell models, these practical classes also involved the generation of radioactive waste, which entails further safety and environmental concerns [5]. To ensure students are still taught these fundamental pharmacological principles and practices [6, 7, 8], we sought to replace these with a more modern method, using NanoLuc Bioluminescence Resonance Energy Transfer (NanoBRET—schematic shown in Figure 1A) [9, 10], a technique that has previously been employed to study GPCRs in undergraduate courses [11]. A three‐part practical class series was devised, using NanoLuc‐tagged β_2_‐adrenoceptors (NLuc‐β_2_AR) expressed in HEK293 cells to measure and calculate ligand affinities (Figure 1B–D, protocol available in Appendix S1—this protocol was developed in line with teaching at the University of Cambridge, and may need adapting for implementation at other institutes of higher learning). In the first session, students calculated the affinity (dissociation constant, *K*_d_) of the fluorescent probe, CA200689, a fluorescent derivative of the antagonist propranolol [12], through a Saturation Binding assay. In the following session, based on their determined dissociation constants (*K*_d_) for CA200689, participants performed a Competition Binding assay to determine the affinity (inhibition constant, *K*_i_) of a range of unlabeled ligands for the receptor. In the final session, the Data Analysis session, students implemented a curve‐fitting spreadsheet to estimate IC_50_ values from the previous dataset to calculate affinities of the unlabeled ligands. We chose the β_2_AR due to the fact that it is one of the 10 most targeted GPCRs, with 45 approved drugs and 4 in clinical trials [4], and due to its relevance to cardiovascular pharmacology, an important module in the taught undergraduate course at the University of Cambridge (both for natural sciences tripos (NST) and medical/veterinary tripos students). NanoBRET was used due to its superior signal‐to‐noise ratio compared to other BRET‐based techniques, enhanced signal stability, and regular implementation in studying ligand binding [13, 14, 15].

## 2 Materials and Methods

### 2.1 Student Participants

Students completing a course in Pharmacology within the second year of study of NST at the University of Cambridge participated in this practical. Data from academic cohorts in 2024 and 2025 were included within this study, with 71 and 61 students from 2024 and 2025, respectively. Students primarily completed the practical in pairs, providing 75 (Saturation Binding) and 78 (Competition Binding) data points across the 2 years. Competition Binding data was also obtained by second‐year students completing a degree in medicine or veterinary sciences during their Mechanism of Drug Action (MoDA) module. 367 students participated over six practical sessions, in groups of 2–3, providing 184 data points for each compound. Data were retained within the cohort from where it originated but was otherwise anonymized. Practical classes are not formally examined in these courses.

### 2.2 Cell Preparation

The NLuc‐β_2_AR construct (pcDNA3.1(+)) was generated using standard restriction enzyme cloning (Dr Sabrina Winfield, University of Cambridge). To enhance surface expression, the signal peptide of the murine 5‐HT_3A_ receptor was positioned upstream of the NLuc [16]. NLuc‐β_2_AR was transfected into HEK293 cells using polyethyleneimine, and positive expression selected for using 800 μg/mL of G418 to generate a stable cell line. Twenty‐four hours prior to the practical, cells were seeded at a density of 10,000 cells/well on white 96‐well plates (Greiner) coated with 0.01% poly‐L‐lysine using a Multidrop Combi Reagent Dispenser (Thermo Fisher Scientific). Cells were cultured in Dulbecco's modified Eagle's medium (DMEM)/F12 Glutamax (Gibco) supplemented with 10% fetal bovine serum (FBS) and 1% antibiotic‐antimycotic (AA) solution, maintained in a 37°C humidified incubator with 5% CO_2_.

### 2.3 Saturation Binding

Students were provided with 20 mL of phosphate‐buffered saline (PBS), 20 mL of PBS supplemented with 0.01% (w/v) of the blocking agent bovine serum albumin (BSA)—PBS‐BSA, 20 μL of CA200689 (HelloBio) at 100 μΜ, and 100 μL of propranolol at 100 μM (Figure 1B). They were first asked to generate 5 mL of PBS‐BSA containing 100 μΜ propranolol, and a PBS‐BSA buffer containing a matching concentration of dimethyl sulfoxide (DMSO). They were then asked to prepare a serial dilution of CA200689 from the starting stock at 100 μM to 0.1 nM. Serial dilutions were then transferred to the ligand plate, diluted in either DMSO‐ or propranolol‐buffer for total and nonspecific binding respectively. Media was removed from the cell plate, and cells washed with PBS, before ligands were transferred to the cell plate. Plates were then given to a demonstrator, who added 0.1 μM NanoGlo substrate (Promega) and then read using a SpectraMax ID 5 multimode microplate reader (Molecular Devices) using a NanoBRET filter module (λ447 nm and λ610 nm long pass).

### 2.4 Competition Binding

Students were provided with 20 mL of PBS, 20 mL of PBS‐BSA, 20 μL of CA200689 at 10 μΜ, and 20 μL of β_2_AR agonists isoprenaline and formoterol at 10 mM and 20 μL of antagonists carvedilol and propranolol at 100 μM (Figure 1C). The students were first asked to generate 8 mL of PBS‐BSA containing CA200689 at a concentration of 2× their determined *K*_d_ value. They were then asked to prepare a serial dilution of each unlabeled ligand from the starting stock to 10 nM (isoprenaline and formoterol) or 0.1 nM (carvedilol and propranolol). Serial dilutions were transferred to the ligand plate and diluted in CA200689‐containing PBS‐BSA buffer. Media was removed from the cell plate, and cells were washed with PBS before ligands were transferred onto the cell plate. Plates were then given to a demonstrator to add 0.1 μM NanoGlo substrate and read using a SpectraMax ID 5 multimode microplate reader (Molecular Devices) using a NanoBRET filter module (λ447 nm and λ610 nm long pass).

### 2.5 Data Analysis and Distribution

Saturation binding experiments were analyzed as part of the competition binding practical, with students asked to plot their datasets to derive the *K*_d_ as determined by the concentration corresponding to half the *B*_max_ value. They were recommended to plot the concentration against the specific binding, using a semi‐logarithmic axis, and interpolate to find the *K*_d_. For our analysis of students' data, affinity values of the labeled and unlabeled ligands were determined with Python (v3.14.0) using the *curve_fit* function from *scipy*'s (1.16.3) *optimize* module. CA200689 *K*_d_ values were determined by fitting a rectangular hyperbole to specific binding curves, calculated as total binding—nonspecific binding, where nonspecific binding was fit with linear regression. As part of the Data Analysis session, students analyzed their data using a bespoke Microsoft Excel spreadsheet, generated in‐house, using the Solver.xlam add‐in. Unlabeled ligand IC_50_ values were determined using the same *curve_fit* function instead using a three‐parameter logistic equation. These IC_50_ values were used to calculate the p*K*_i_, via the Cheng‐Prusoff correction. For statistical comparisons, the distributions of students' logged‐*K*_d_ and ‐*K*_i_ values from each practical class were assessed using the Shapiro–Wilk test and D'Agostino *K* ^2^ test. Normal distributions were compared to a single value using a one‐sample *t*‐test, while non‐normal distributions were compared to a single value using a Wilcoxon signed‐rank test. Normal distributions were compared to each other using Student's *t*‐test, while non‐normal distributions used the Mann–Whitney *U* test. Root mean squared deviations (RMSD) between experimental points and fitted points were calculated as an indirect measure to evaluate how much students' experimental data resembled expected results. To compare across student groups, RMSD values were normalized as a percentage of the mean (nRMSD). Data visualizations for figures were created using GraphPad Prism 10.

### 2.6 Student Feedback Survey

Fifty‐two students from the NST 2nd‐year pharmacology practical course 2025 completed an online survey to communicate their experience in the practical series. The survey was completely anonymized, and students were given a disclaimer prior to completion of the survey that outlined the purpose of the survey and communicated that all results would be anonymous and have no bearing on course marking or record of attendance, nor was completion mandatory. Students were required to agree to the terms of this disclaimer before being provided with the survey. The survey consisted of eight close‐ended, unipolar Likert scale items. Students were given the choice of “Strongly Disagree,” “Disagree,” “Neutral,” “Agree,” and “Strongly Agree” for each item. Answering all items was required for full completion of the survey. The Likert scale items were designed to assess student opinion regarding administration of the practical, relevance of the practical to lecture theory, support of their understanding of the theory, relevance of techniques used in the practical, and reduction of animal use in teaching laboratory settings. The survey was administered after completion of the data analysis segment of the three‐part practical series and took approximately 10 min.

### 2.7 Nomenclature of Targets and Ligands

Key protein targets and ligands in this article are hyperlinked to corresponding entries in http://www.guidetopharmacology.org and are permanently archived in the Concise Guide to PHARMACOLOGY2025/26 [17].

## 3 Results

**FIGURE 2:**
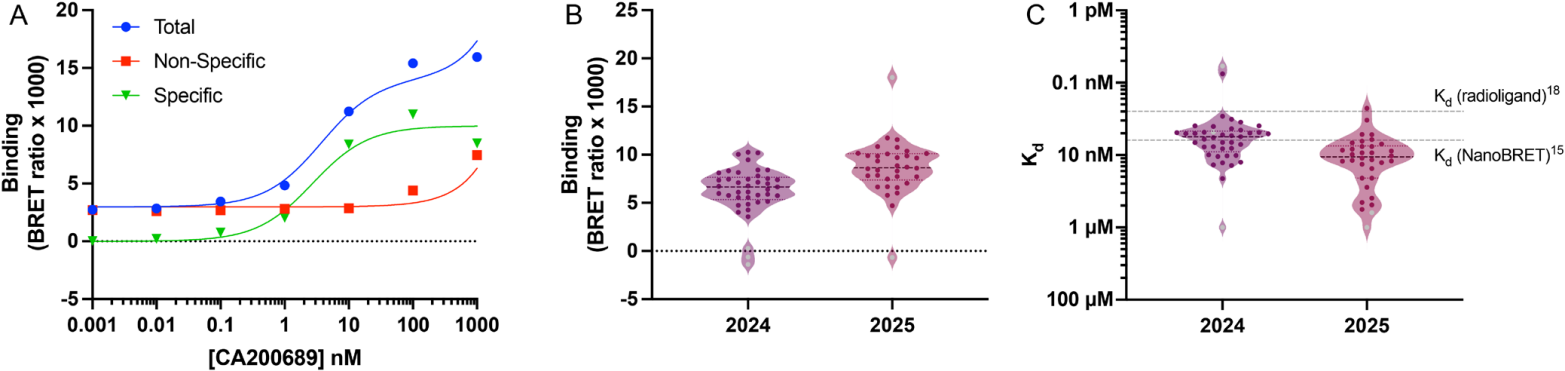
Determining fluorescent ligand affinity using Saturation Binding. (A) Example student data for Total, Nonspecific, and the calculated Specific binding. (B) *B*_max_ values from both cohorts. (C) CA200689 affinity (*K*_d_) determined across 2024 and 2025 cohorts, with dotted lines to indicate literature affinities determined with radioligand [18] and NanoBRET [15]. *K*_d_ and *B*_max_ values from datasets where the values are not realistic (*K*_d_ ≥ 1 μM or *B*_max_ ≤ 1) are highlighted in gray in (B), (C).

This practical class series was completed by second‐year undergraduate students, reading for a degree in the NST. Two academic cohorts (2024 and 2025) were included within this study to test improvements made to the protocol to increase robustness. Students were asked to perform a NanoBRET Saturation Binding experiment (Figure 1B), determining the affinity of the fluorescent derivative of propranolol, CA200689, for the NLuc‐tagged β_2_AR. Cells were treated with a range of concentrations of CA200689, in the absence and presence of unlabeled propranolol to measure total and nonspecific binding, respectively (Figure 2A–C). *K*_d_ values reported in nM were calculated from the two cohorts (Figure 2C). The mean affinity from the 2024 cohort was 3.77 nM, which increased to 15.85 nM for the 2025 cohort, potentially due to the increase in unlabeled propranolol concentration, from 100 nM in 2024 groups to 1 μM in 2025, reducing the degree of nonspecific binding at high concentrations of CA200689. The affinity from the 2024 cohort showed excellent agreement with the published affinity determined through NanoBRET (3.9 nM) [15] (Wilcoxon signed‐rank test, *p* = 0.608). The 2025 cohort's dataset better displayed saturable binding, with a decreased mean nRMSD compared to the 2024 cohorts' data (Table 1) when fit using a rectangular hyperbola. The reduced residual error is likely a result of the reduced nonspecific binding. Of the 75 datasets from across both years, only five datasets displayed either no affinity (*K*_d_ ≥ 1 μM) or insufficient maximal binding (*B*_max_ ≤ 1) to appropriately calculate an affinity, meaning over 90% of students were successful in performing the saturation binding assay (Figure 2A–C).

**TABLE 1:** *K*_d_ values for CA200689 determined through saturation NanoBRET binding. Student data are reported as geometric mean (SD factor) in nM.

	*K*_d_ (computational)	*K*_d_ (graphical)
Literature	3.9 (NanoBRET [15])	N/A
NST 2024	3.77 (4.93)	6.09 (2.54)
NST 2025	15.85 (5.58)	11.81 (2.25)

The following week students were asked to determine an affinity for the fluorescent ligand by plotting the data by hand and visually estimating a *K*_d_ value (Table 1). The students' graphically determined *K*_d_ values were significantly different when compared to *K*_d_ values calculated computationally from their data (two‐sided Mann–Whitney *U* test, *p* = 0.001), likely due to inaccurate fitting of sigmoidal curves or rounding when interpolating the data (indeed, the modal determined *K*_d_ by the students was 10 nM). The 2024 cohort's affinity was still lower than that determined by the 2025 cohort (Table 1), consistent with the computational analysis. After graphically determining a *K*_d_ value from their data, students were asked to perform a Competition Binding experiment for the agonists isoprenaline and formoterol and antagonists carvedilol and propranolol (Figure 1C). The assay was performed using a concentration of CA200689 equal to their calculated *K*_d_ or 10 nM when their calculated affinity was below 5 nM or above 15 nM (to ensure a reasonable assaying window and conserve resources, respectively).

**FIGURE 3:**
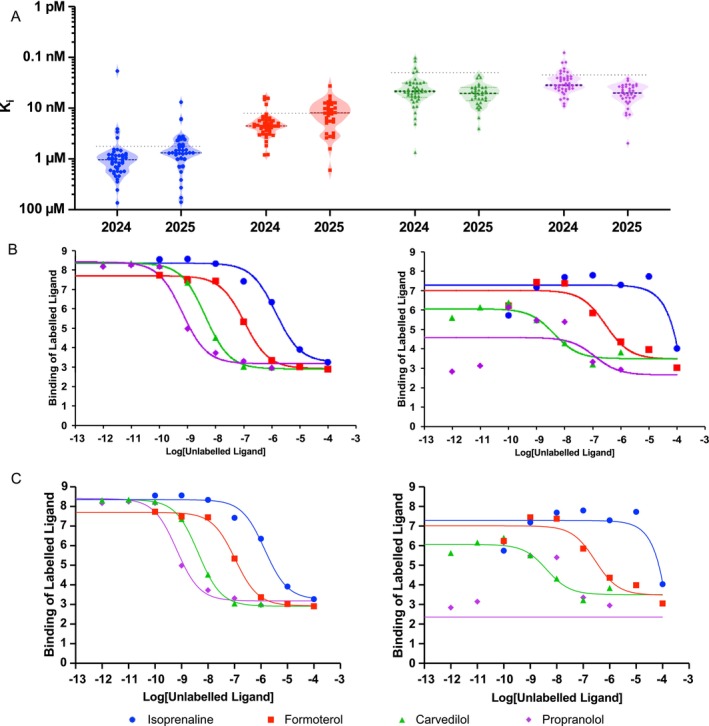
Affinity of unlabeled ligands determined through Competition Binding. (A) *K*_i_ values for the four unlabeled ligands, from the 2024 and 2025 cohorts. Gray dotted lines indicate literature affinity values determined through similar techniques. Example competition binding datasets, plotted and fit with a three‐parameter logistic equation in (B) Microsoft Excel, as per the Data Analysis session, or (C) GraphPad Prism. The same datasets are used for both analysis methods to highlight their similarities.

The mean determined *K*_i_ values for the unlabeled ligands are listed in Table 2. Within the NST 2025 cohort, isoprenaline and formoterol produced affinities that were nonsignificantly different to the literature values determined through time‐resolve fluorescence resonance energy transfer [18]. The affinities determined for propranolol and carvedilol are approximately 10‐fold lower than literature reports [15, 19] (Figure 3A). This may be due to the techniques via which these values have been determined (i.e., live cell‐based assays compared to prepared membranes), or binding to the unlabeled endogenous adrenoceptors expressed in the HEK293 cells.

**TABLE 2:** Affinities (*K*_i_) for unlabeled ligands from literature datasets compared to undergraduate cohorts. Student data are reported as geometric mean (SD factor) in nM.

	Isoprenaline	Formoterol	Carvedilol	Propranolol
Literature	398 [18]	15.8 [18]	0.40 [19]	0.49 [15]
NST 2024	1098 (5.83)* *p* < 0.0001	48.75 (3.02)* *p* < 0.0001	2.28 (4.31)* *p* < 0.0001	1.02 (2.68)* *p* < 0.0001
NST 2025	621.8 (5.71), ns *p* = 0.08	22.54 (4.59), ns *p* = 0.51	3.09 (2.73)* *p* < 0.0001	3.12 (3.15)* *p* < 0.0001
MoDA	506.3 (31.3)* *p* = 0.0073	62.07 (14.82)* *p* < 0.0001	2.86 (10.26)* *p* < 0.0001	2.43 (11.24)* *p* < 0.0001

*Note:* Significant difference from literature values determined using Wilcoxon signed‐rank test.

Abbreviation: ns, nonsignificant.

* *p* < 0.05.

For the final session of the three‐part practical, the students analyzed their own Competition Binding dataset, as part of the Data Analysis practical class (Figure 1D). Students calculated average NanoBRET ratios (multiplied by 1000 for ease of handling) and imported these values to a custom Microsoft Excel spreadsheet developed for curve fitting (available for download at https://github.com/Theo‐BRN/Competition_Binding_Excel, example outputs compared to GraphPad Prism in Figure 3B,C highlighting the similar ability of Micrsosoft Excel and GraphPad Prism to interpret datasets of differing qualities). Data were manually fitted in the practical class by adjusting “Top,” “Bottom,” and “Log(IC_50_)” values to reduce the “Objective Value.” Following manual manipulation, a “best‐fit” solution was identified using the Solver Add‐in, reducing the “Objective Value” by minimizing the variable parameters. The IC_50_ value was then used to calculate the *K*_i_, using the Cheng‐Prusoff correction.

**FIGURE 4:**
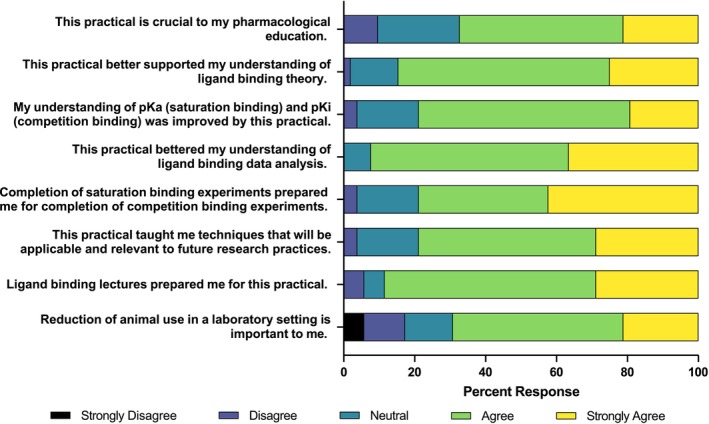
Student Feedback for the ligand binding practical series is overwhelmingly positive. Percentage of responses corresponding to each Likert scale ranking for eight questions asked immediately following the Data Analysis session.

Student feedback was obtained through use of an anonymous survey, completed immediately following the Data Analysis session of the three‐part practical (Figure 4). Feedback encompassed all parts of the practical and was completed by 52 students from the 2025 NST pharmacology cohort, and was based on personal evaluation of their own experience, as practical classes are not examined so improvement in performance could not be assessed. The overall feedback from the survey conveyed a positive learning experience of the students with over 80% agreeing that this series of practical classes improved their understanding of ligand binding theory and analysis (“Agree” or “Strongly Agree”). Students also agreed that completion of week 1 (“Saturation Binding”) enhanced their experience in week 2 (“Competition Binding”), meaning the improved data acquisition observed was also experienced by the students. The least positive feedback obtained surrounded the students' views on reducing animal use in practical classes (30.8% ranked “Neutral” or lower) or the importance of this practical on their pharmacological education (32.7% ranked “Neutral” or lower), but in each case over 67% of students still had a positive response. This suggests that this practical is received well by the students.

**FIGURE 5:**
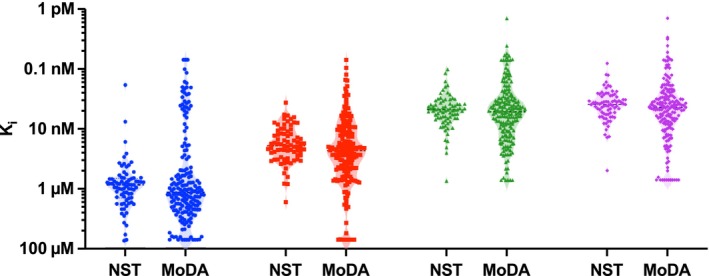
Expansion of the Competition Binding Session to the Medical Sciences Tripos students who study MoDA. Unlabeled ligand affinities (*K*_i_) comparing the NST (combined 2024 and 2025 year groups) and MoDA student cohorts.

Following the success of this practical for the NST students, and the importance of studying ligand binding in pharmacodynamics, this practical was performed with the larger cohort of students studying MoDA as part of the second‐year medical and veterinary sciences tripos. Due to the larger group size (~350 students) and temporal and financial considerations, these students only completed the Competition Binding and Data Analysis components. They were therefore provided with a pre‐determined concentration of 10 nM CA200689 to use. There was a higher variability on this dataset, which could be attributed to the omission of the Saturation Binding practical class, meaning the students were less familiar with the techniques involved (Figure 5).

## 4 Discussion

To maintain practical teaching of the measurement of drug‐receptor interactions and calculate affinity values, the traditional radioligand binding was replaced with a NanoBRET ligand binding practical series. This class series was spread across 3 weeks—determining the affinity of the fluorescent probe via Saturation Binding, determining the affinity of unlabeled agonists and antagonists via Competition Binding, and using model fitting and parameter estimation as part of a Data Analysis practical. The primary goal was to ensure students still obtained practical experience determining ligand affinities to support the taught elements of their course, which was achieved based on the results of the feedback survey. Whilst practical classes are not examined at this institution, theoretical knowledge of key pharmacological concepts (such as drug affinity) can be challenging to comprehend without application within a practical setting [7, 8].

This class provides key skills in experimental techniques, in particular the use of in vitro techniques that require precision and accuracy. Through implementation of a protocol, with additional questions as to the importance of different stages, for example, measuring saturation binding in the absence and presence of unlabeled propranolol, and the importance of keeping DMSO concentrations constant (Appendix S1), students are also provided with core knowledge in methodological principles in addition to the core skill of good experimental design. NanoBRET has previously been shown suitable for use in undergraduate classes, for measuring β‐arrestin recruitment [11]. Herein we use a fluorescent ligand to measure binding affinities of unlabeled agonists and antagonists, highlighting the versatility of the technique to GPCR research and taught practical classes. Our hope is that by demonstrating the suitability of NanoBRET for application in undergraduate classes, other institutions might be persuaded to adopt animal‐free methodologies that are not solely computational [20].

An important aspect of this practical class involved the introduction of students to computational analysis of data, using an Excel spreadsheet with the Solver function for parameter estimation. At the University of Cambridge, data interpretation within examinations relies on visual estimation from hand drawn graphs, and as such the students are less familiar with mathematical approaches used by research groups. Inaccuracy from visual estimation is highlighted by the differences in *K*_d_ values for CA200689 estimated by students compared to those determined computationally in our analysis. We further highlight the suitability of this program, providing similar outputs to software such as GraphPad Prism, which is not commonly available to undergraduate students, for datasets of different qualities.

There are some potential limitations for this practical. Whilst we were able to successfully implement this practical to a large student cohort (approximately 350 students on the MoDA course), this is accompanied by a time‐consuming set up, with cells needing to be seeded 24 h prior using a Multidrop Combi reagent dispenser. Additionally, to read all completed plates, three plate readers were used for a group size of ~50 students working in pairs. This represents a financial burden if equipment is otherwise not available within the research institution. These are, however, considerations for large student cohorts—for smaller cohorts these limitations are less pressing, with only one plate reader used for group sizes of up to 40 in 2024, with all plates able to be read within a three‐hour class. However, through use of a stable cell line expressing NLuc‐β_2_AR, the amount of preparatory work required is reduced relative to other NanoBRET techniques [11]. These assays are also relatively stable, with signal maintained for 180 min [21] following the addition of luciferase substrate, meaning a large number of plates can still be recorded with fewer plate readers, without loss of data.

## Author Contributions

**Abigail Pearce:** writing – original draft, writing – review and editing, investigation, validation, conceptualization, resources, visualization. **Xianglin Huang:** writing – review and editing, investigation, validation, resources, methodology. **Theo Redfern‐Nichols:** software, writing – review and editing, conceptualization, supervision, resources, data curation, writing – original draft. **Claudia M. Sisk:** writing – review and editing, formal analysis. **Dewi Safitri:** investigation, validation, resources, writing – review and editing. **Michael Collins:** data curation, writing – review and editing. **Milena A. Malcharek:** data curation, writing – review and editing. **Sergio Tomey García:** supervision, writing – review and editing. **Chiharu Nakamura:** supervision, writing – review and editing. **Andrzej Harris:** conceptualization, supervision, formal analysis, writing – review and editing. **Graham Ladds:** conceptualization, supervision, resources, writing – review and editing, funding acquisition, project administration.

## Funding

Funds for establishing this practical class were awarded from the Marmaduke Sheild Fund. Work in G.L.'s lab is funded by the BBSRC (BB/Y513817/1 and BB/W014831/1, to A.P.). M.A.M. and T.R.‐N. were funded by BBSRC‐iCase studentships with AstraZeneca (BB/X010899/1 to M.A.M. and BB/V509334/1 to T.R.‐N.). M.C. is funded by a BBSRC‐iCase studentship with Syngenta (BB/Z516454/1). X.H. was co‐funded by China Scholarship Council and Cambridge Trust. C.M.S. was funded by a Cambridge Trust International Scholarship in conjunction with the Gonville and Caius Stanley Elmore PhD Studentship. M.C. and D.S. were funded through the David James Trust. G.L. is a Royal Society Industry Fellow (INF/R2/212001).

## Conflicts of Interest

The authors declare no conflicts of interest.

## Supporting information


**Appendix S1:** Ligand binding assay protocol.

## Data Availability

The data that support the findings of this study are available from the corresponding author upon reasonable request. Some data may not be made available because of privacy or ethical restrictions. The Microsoft Excel spreadsheet for data analysis is available to download at https://github.com/Theo‐BRN/Competition_Binding_Excel.
