# Supplementary material for: Using a NanoBRET‐Based Ligand‐Binding Assay at the β2‐Adrenoceptor for Undergraduate Pharmacology Education
Source: Pharmacol Res Perspect. 2026 Jun 9;14(3):e70275. doi: 10.1002/prp2.70275 (PMC13248772; doi:10.1002/prp2.70275)
Supplement: Supplementary file 1 — Appendix S1: Ligand binding assay protocol. [file PRP2-14-e70275-s001.docx]

**Ligand binding assay protocol**

**Determining the binding affinity of fluorescent and unlabelled ligands at the β2-adrenoceptor (β2AR) using bioluminescence resonance energy transfer (BRET).**

**Background**

One of the most important properties of a drug is the affinity with which it binds to its target. Traditionally, binding affinity has been determined through the use of radiolabelled ligands. However, the development of NanoBRET and small fluorophores that can be conjugated to ligands has allowed us to move away from using radioactive substances. Bioluminescence Resonance Energy Transfer (BRET) is a technique that exploits energy transfer from a donor luciferase to an acceptor fluorophore when the two are in close proximity.

For a NanoBRET binding assay, the GPCR is tagged with NanoLuc (Nluc), a small 19 KDa luciferase from the deep-sea shrimp, and the ligand conjugated to a red fluorophore acceptor. Nluc produces light at 460nm by oxidising the substrate furimazine. This emission excites the fluorescent ligand through BRET, only when it is bound to the GPCR. This excitation of the fluorescent ligand will subsequently lead to emission of light at 647 nm. The BRET ratio (647 nm emission/460 nm emission) can then be calculated to quantify ligand binding. NanoBRET experiments allow real-time quantitative analysis of the pharmacology of ligand-receptor interactions in living cells and can be used to determine binding affinity of labelled and unlabelled ligands through saturation and competition experiments, respectively.

In the next two practical sessions, you will use a fluorescent ligand to the β2 adrenoceptor (β2AR) to determine the binding affinity of a selection of β2AR ligands. The fluorescent ligand, CA200689 (referred to as CA throughout), is a high affinity β2AR antagonist containing a polyamide linker connected to the BY630 fluorophore (Baker *et al.,* 2011). Using a cell line expressing an N-terminally Nluc-tagged β2AR, binding assays can be conducted.


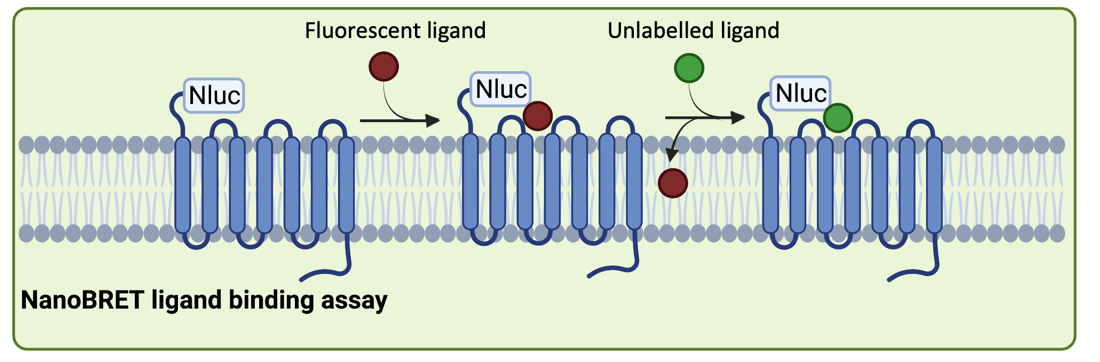


**Week 1**

You will perform a saturation binding experiment to determine the binding affinity (Kd) of the CA compound. HEK293 cells stably expressing Nluc-β2AR will be treated with increasing concentrations of CA. This is done in the absence (to determine total binding) and presence (to determine non-specific binding) of a saturating concentration of a high-affinity unlabeled β2AR antagonist, propranolol. Specific binding can then be calculated and Kd for the fluorescent ligand determined.

**Week 2**

You will calculate the Kd of CA, determined from the first practical. Then, you will use a concentration of CA equal to the Kd, to investigate the binding affinities of different β2AR ligands in a competition binding experiment. Cells will be incubated with a fixed concentration of CA (Kd concentration) with increasing concentrations of unlabelled ligands, which will dose-dependently displace CA binding leading to a loss of BRET.

**Week 3**

Using your data from week 2, you will calculate the binding affinity of the unlabelled ligands (Ku) using the Cheng and Prusoff equation and some excel-based curve fitting software.

Papers:

Stoddart *et al.,* 2015 Application of BRET to monitor ligand binding to GPCRs

Sykes *et al.,* 2019 Binding kinetics of ligands acting at GPCR

Suchankova, A., Harris, M., & Ladds, G. (2021). Measuring the rapid kinetics of receptor-ligand interactions in live cells using NanoBRET. In *Methods in Cell Biology* (Vol. 166, pp. 1-14). Academic Press.

**Week 1: Saturation binding – Determining K_d_ of the fluorescent ligand**

Overview

In this part of the assay, you will prepare two stock solutions of PBS-BSA: one containing DMSO and one propranolol, which will be added to the ligand plate. Separately, CA will be serially diluted in DMSO in eppendorf tubes and then transferred to the solutions in the ligand plate. At this point you will receive the cell plate. The media in the wells is removed and the wells washed with PBS. The different dilutions of CA are added from the ligand plate according to the map. A demonstrator will then add Nluc substrate before reading the plate.

1/ Prepare buffers containing DMSO or propranolol

You will be provided with PBS buffer containing 0.1% BSA (labeled PBS-BSA).

You have also been given a stock of 1 x 10^-4^ M propranolol dissolved in 100% DMSO.

Label 2 falcon tubes – one with “DMSO-Buffer” and one with “Propranolol-Buffer”.

Make up 5ml of buffer containing 1 x 10^-6^ M propranolol and a control buffer with equivalent percentage DMSO, both in PBS-BSA. Invert the solutions to mix (do not shake).

Q: What is the volume of 1 x 10^-4^ M propranolol that you will need to add to make 5ml “Propranolol-buffer” containing 1 x 10^-6^ M propranolol? What is the volume of DMSO that you need to add to “DMSO-buffer” to keep %DMSO consistent?

|  |
| --- |

Q: Why do we need to measure binding with and without unlabelled propranolol?

|  |
| --- |

2/Before diluting the ligands

We want to test the following final concentrations of CA in our binding assay with and without 1 x 10^-6^ M propranolol:

- 1 x 10^-6^ M (1)
- 1 x 10^-7^ M (2)
- 1 x 10^-8^ M (3)
- 1 x 10^-9^ M (4)
- 1 x 10^-10^ M (5)
- 1 x 10^-11^ M (6)
- 1 x 10^-12^ M (7)

In all wells of our assay plate, we want the final % DMSO to be constant.

Q: Why is it important that all wells have the same final % DMSO? And why should this be a low %?

|  |
| --- |

3/ Preparing ligand dilutions

You have been provided with a stock solution of 30 μl of CA200689 (CA) at 1 x 10^-4^ M.

Serially dilute CA (concentrations A-E) in 100% DMSO in eppendorfs (up to 20 ul per tube):

- 1 x 10^-4^ M (A)
- 1 x 10^-5^ M (B)
- 1 x 10^-6^ M (C)
- 1 x 10^-7^ M (D)
- 1 x 10^-8^ M (E)
- 1 x 10^-9^ M (F)
- 1 x 10^-10^ M (G)

Mix with pipette when diluting. Also, please leave the tubes in the eppendorf rack if possible or wrap them with aluminium foil to protect CA from light.

Now you are ready to load your dilution plate according to the scheme below.

- Add 245 µl of DMSO-buffer or propranolol-buffer to each well (pipetting the large volume first improves accuracy).
  - Use DMSO buffer in non-starred wells and propranolol buffer in starred wells (*).
  - Your wells will be very full, so be careful with the ligand plate!
- Add 5 µl of A-G in appropriate wells.
- Add 5 µl 100% DMSO to the appropriate wells.

| Ligand plate plan | | | | | | | | | | | | |
| --- | --- | --- | --- | --- | --- | --- | --- | --- | --- | --- | --- | --- |
|  | 1 | 2 | 3 | 4 | 5 | 6 | 7 | 8 | 9 | 10 | 11 | 12 |
| A | A | *A |  |  |  |  |  |  |  |  |  |  |
| B | B | *B |  |  |  |  |  |  |  |  |  |  |
| C | C | *C |  |  |  |  |  |  |  |  |  |  |
| D | D | *D |  |  |  |  |  |  |  |  |  |  |
| E | E | *E |  |  |  |  |  |  |  |  |  |  |
| F | F | *F |  |  |  |  |  |  |  |  |  |  |
| G | G | *G |  |  |  |  |  |  |  |  |  |  |
| H | DMSO | *DMSO |  |  |  |  |  |  |  |  |  |  |

Wrap the ligand plate with aluminium foil to protect the CA compound from light.

4/ Adding ligands to the cell plate

Ask a demonstrator for a cell plate

There are cells coating the bottom of each well, which you must be careful not to disrupt – pipette from the corner of the well and add liquid down the wall of the well.

- - - Pipette to gently take all of the medium out of each well (100μl). Put liquid waste in liquid waste pot.
    - Add 100ul of PBS into each well and gently remove with pipette.
    - Add 100μl prepared ligands from dilution plate to cell plate and cover with aluminium foil.
    - You will need to add in duplicates, as per the diagram.
      - Wells containing propranolol are denoted with a star (*).

| Final Cell plate plan | | | | | | | | | | | | |
| --- | --- | --- | --- | --- | --- | --- | --- | --- | --- | --- | --- | --- |
|  | 1 | 2 | 3 | 4 | 5 | 6 | 7 | 8 | 9 | 10 | 11 | 12 |
| A | 1 | 1 | *1 | *1 |  |  |  |  |  |  |  |  |
| B | 2 | 2 | *2 | *2 |  |  |  |  |  |  |  |  |
| C | 3 | 3 | *3 | *3 |  |  |  |  |  |  |  |  |
| D | 4 | 4 | *4 | *4 |  |  |  |  |  |  |  |  |
| E | 5 | 5 | *5 | *5 |  |  |  |  |  |  |  |  |
| F | 6 | 6 | *6 | *6 |  |  |  |  |  |  |  |  |
| G | 7 | 7 | *7 | *7 |  |  |  |  |  |  |  |  |
| H | DMSO | DMSO | *DMSO | *DMSO |  |  |  |  |  |  |  |  |

At this point you will need to label the lid and side of the cell plate with your crsid and give your plate to a demonstrator. They will add 100μl of Nluc substrate to each well, and incubate for 10-minutes. They will then read the plate using the SpectraMax ID plate reader, measuring the emission at 460nm and using a 610nm long pass filter.

Data will be given back in the next week for calculating the Kd of CA200689, which will be used in the competition assay.

**Week 2: Competition binding - Determining binding affinity of unlabelled ligands**

Overview

In the second part of this practical, you will first need to plot your data from last week to calculate the K_d_ for CA. You will then create one stock solution of PBS-BSA containing CA at 2xK_d_ which will be added to the appropriate wells of the ligand plate. Separately, each of the 4 unlabelled ligands will be serially diluted in DMSO in eppendorf tubes and then transferred to the solution in the ligand plate. At this point you will receive the cell plate. The media in the wells is removed, and the different dilutions of ligands added from the ligand plate according to the map. A demonstrator will then add Nluc substrate before reading the plate.

1/ Calculate the K_d_ of CA200689

We will start off this practical by calculating the K_d_ of CA from your data generated in the saturation binding assay.

Check your K_d_ value with a demonstrator

|  |
| --- |

2/ Prepare CA200689 (CA) buffer

You are provided with 20 μl of 1 x 10^-5^ M CA and 20ml PBS-BSA buffer.

You need to prepare 8 ml of buffer with 2 x K_d_ CA in PBS-BSA. Wrap the falcon tube in foil as CA is light sensitive.

Q: What volume of stock CA will you add to what volume of PBS/BSA?

|  |
| --- |

3/ Before ligand dilution

You are provided with 30 µl of stock solutions of:

- 1 x 10^-2^ M Isoprenaline (Iso)
- 1 x 10^-2^M Formoterol (For)
- 1 x 10^-4^ M Carvedilol (Car)
- 1 x 10^-4^ M Propranolol (Pro)

We will be testing all four compounds, in duplicates, at final concentrations of:

- For Isoprenaline (Iso) and Formoterol (For)
- 1 x 10^-4^ M (1)
- 1 x 10^-5^ M (2)
- 1 x 10^-6^ M (3)
- 1 x 10^-7^ M (4)
- 1 x 10^-8^ M (5)
- 1 x 10^-9^ M (6)
- 1 x 10^-10^ M (7)

For Carvedilol (Car) and Propranolol (Pro):

- 1 x 10^-6^ M (1)
- 1 x 10^-7^ M (2)
- 1 x 10^-8^ M (3)
- 1 x 10^-9^ M (4)
- 1 x 10^-10^ M (5)
- 1 x 10^-11^ M (6)
- 1 x 10^-12^ M (7)

4/ Preparing ligand dilution

Dilute in eppendorf tubes in 100% DMSO

- Prepare dilutions A-G for Isoprenaline (Iso), Formoterol (For), Carvedilol (Car) and Propranolol (Pro) in eppendorfs.
- Use 18μL of DMSO and 2μL of ligand for the serial dilutions

For Isoprenaline and Formoterol

- 1 x 10^-2^ M (A)
- 1 x 10^-3^ M (B)
- 1 x 10^-4^ M (C)
- 1 x 10^-5^ M (D)
- 1 x 10^-6^ M (E)
- 1 x 10^-7^ M (F)
- 1 x 10^-8^ M (G)

For Carvedilol and Propranolol

- 1 x 10^-4^ M (A)
- 1 x 10^-5^ M (B)
- 1 x 10^-6^ M (C)
- 1 x 10^-7^ M (D)
- 1 x 10^-8^ M (E)
- 1 x 10^-9^ M (F)
- 1 x 10^-10^ M (G)

Add 245 µl CA buffer to each well.

Add 5 µl of relevant dilution to each well as per the plan below.

Wrap the plate with aluminium foil to protect the CA compound from light.

| Ligand dilution plate plan | | | | | | | | | | | | |
| --- | --- | --- | --- | --- | --- | --- | --- | --- | --- | --- | --- | --- |
|  | 1 | 2 | 3 | 4 | 5 | 6 | 7 | 8 | 9 | 10 | 11 | 12 |
| A |  |  |  |  | (A) Iso | (A) For | (A) Car | (A) Pro |  |  |  |  |
| B |  |  |  |  | (B) Iso | (B) For | (B) Car | (B) Pro |  |  |  |  |
| C |  |  |  |  | (C) Iso | (C) For | (C) Car | (C) Pro |  |  |  |  |
| D |  |  |  |  | (D) Iso | (D) For | (D) Car | (D) Pro |  |  |  |  |
| E |  |  |  |  | (E) Iso | (E) For | (E) Car | (E) Pro |  |  |  |  |
| F |  |  |  |  | (F) Iso | (F) For | (F) Car | (F) Pro |  |  |  |  |
| G |  |  |  |  | (G) Iso | (G) For | (G) Car | (G) Pro |  |  |  |  |
| H |  |  |  |  | DMSO |  |  |  |  |  |  |  |

5/Adding ligands to the cell plate

Ask a demonstrator for cell plates

There are cells in the plates so be careful not to disrupt the cell layer – pipette from the corner of the well and add liquid down the wall of the well.

- - - Pipette to gently remove all of the medium out of each well (100μl). Put liquid waste in liquid waste pot.
    - Add 100ul of PBS into each well and gently remove them with pipette.
    - Add 100μl of the prepared ligands from the dilution plate to cell plate and cover with aluminium foil.

You will need to add in duplicates, as per the diagram.

| Final Cell Plate Plan | | | | | | | | | | | | | |
| --- | --- | --- | --- | --- | --- | --- | --- | --- | --- | --- | --- | --- | --- |
|  | 1 | 2 | 3 | 4 | 5 | 6 | 7 | 8 | 9 | 10 | 11 | 12 |  |
| A |  |  |  |  | 1 Iso | 1 Iso | 1 For | 1 For | 1 Car | 1 Car | 1 Pro | 1 Pro |  |
| B |  |  |  |  | 2 Iso | 2 Iso | 2 For | 2 For | 2 Car | 2 Car | 2 Pro | 2 Pro |  |
| C |  |  |  |  | 3 Iso | 3 Iso | 3 For | 3 For | 3 Car | 3 Car | 3 Pro | 3 Pro |  |
| D |  |  |  |  | 4 Iso | 4 Iso | 4 For | 4 For | 4 Car | 4 Car | 4 Pro | 4 Pro |  |
| E |  |  |  |  | 5 Iso | 5 Iso | 5 For | 5 For | 5 Car | 5 Car | 5 Pro | 5 Pro |  |
| F |  |  |  |  | 6 Iso | 6 Iso | 6 For | 6 For | 6 Car | 6 Car | 6 Pro | 6 Pro |  |
| G |  |  |  |  | 7 Iso | 7 Iso | 7 For | 7 For | 7 Car | 7 Car | 7 Pro | 7 Pro |  |
| H |  |  |  |  | DMSO | DMSO |  |  |  |  |  |  |  |

At this point you will need to label the cell plates with your crsid and the Kd you used for CA, then give your plate to a demonstrator. They will add 100μl of Nluc substrate to each well and incubate for 10-minutes. They will then read the plate using the SpectraMax ID plate reader, measuring the emission at 460nm and using a 610nm long pass filter.

Data will be given back in the next week in the binding analysis section
